# Prevalence of and coping mechanisms against mental and psychological burnout among healthcare professionals in a Malaysian public hospital: A cross-sectional study

**DOI:** 10.51866/oa.755

**Published:** 2025-04-18

**Authors:** Nuratikah Adillah Rezuan, Aina Amanina Abdul Jalil, Zakiah Mohd Noordin

**Affiliations:** 1 BPharm, MClin Pharm, Faculty of Pharmacy and Health Sciences, Royal College of Medicine Perak, Universiti Kuala Lumpur, Ipoh, Perak, Malaysia. Email: aina.amanina@unikl.edu.my; 2 BPharm, Faculty of Pharmacy and Health Sciences, Royal College of Medicine Perak, Universiti Kuala Lumpur (RCMP UniKL), Ipoh, Perak, Malaysia.; 3 BPharm, MClin Pharm, Department of Pharmacy Practice and Clinical Pharmacy, Faculty of Pharmacy, Universiti Teknologi MARA (UiTM) Selangor Branch, Puncak Alam Campus, Puncak Alam, Selangor, Malaysia.; 4 Cardiology Therapeutics Research Group, Faculty of Pharmacy, Universiti Teknologi MARA Selangor Branch, Puncak Alam Campus, Selangor, Malaysia.

**Keywords:** Burnout, Healthcare professionals, Coping, Mental health

## Abstract

**Introduction:**

Burnout is a significant issue among healthcare professionals, primarily including doctors, nurses and pharmacists. This study aimed to identify the factors contributing to burnout and the coping mechanisms employed by healthcare professionals working in a public hospital in Malaysia.

**Methods:**

This study used an analytic observational method with a cross-sectional design, collecting data via online and printed questionnaires. Healthcare professionals from Hospital Raja Permaisuri Bainun were recruited through non-probability convenience and snowball sampling. Descriptive statistics were utilised to analyse the prevalence of and coping mechanisms against mental and psychological burnout, while Pearson’s chi-square and inferential statistics were used to identify the association between burnout and socio-demographic factors such as age, sex and educational level, with P-values of <0.05 indicating statistical significance.

**Results:**

Approximately 54.7% of the participants had moderate burnout. The majority experienced personal and work-related burnout (81.9%) and patient-related burnout (85.5%). Significant associations were found between burnout and age (P<0.001). Religion was the most commonly utilised coping mechanism by the participants.

**Conclusion:**

The results underscore the urgent need for government institutions to implement targeted interventions and training programmes aimed at addressing burnout among healthcare professionals. Focusing on these issues can enhance mental health support, improve job satisfaction and ultimately ensure better patient care outcomes.

## Introduction

Healthcare is one of the most demanding professions, requiring high mental and psychological resilience to cope with its challenges. Nordin et al. found that hospital-based healthcare professionals were at a higher risk of burnout and stress compared to professionals working in peripheral healthcare services, which encompass both clinical and public health sectors.^1^ In Malaysia, hospital workers often endure long shifts and frequent on-calls, while clinic workers generally adhere to standard working hours with less intense on-call responsibilities.^2^

Burnout, as defined by the World Health Organization in 2019, is a chronic condition resulting from prolonged workplace stress.^3^ The 11th International Classification of Diseases describes burnout as a professional phenomenon characterised by fatigue and reduced efficacy due to job-related stress.^4^ Persistent stress can lead to elevated levels of cortisol, a primary stress hormone, which may result in long-term adverse effects on mental health if not addressed.^5^

Burnout is a multifaceted phenomenon that can be classified into several dimensions, primarily based on the Maslach Burnout Inventory, which assesses three core components: emotional exhaustion, depersonalisation and reduced personal accomplishment. Emotional exhaustion refers to feelings of being emotionally drained and depleted, while depersonalisation involves a sense of detachment or cynicism towards patients and colleagues. Conversely, reduced personal accomplishment reflects a decline in feelings of competence and achievement at work. The classification of burnout severity can vary, with individuals categorised as experiencing low, moderate or high levels of burnout based on their scores across these dimensions. For instance, high emotional exhaustion and depersonalisation scores, combined with low personal accomplishment scores, indicate severe burnout.^6-8^ Additionally, Kristensen et al. expanded the classification by introducing personal burnout, work-related burnout and client-related burnout, emphasising the need for a nuanced understanding of how these factors interact and contribute to overall burnout experiences among healthcare professionals.^9^

The study conducted by Roslan et al. involving 893 healthcare professionals in Malaysia highlighted significant burnout levels. More than half of their respondents experienced personal burnout; 39.1% reported work-related burnout; and 17.4% attributed their burnout to patient-related factors. The study also found that personal burnout was highest among pharmacists and medical laboratory technologists and that work-related burnout was prevalent among health inspectors and medical laboratory technologists. Conversely, patient-related burnout was most common among paramedics and doctors.^10^

Globally, burnout prevalence varies widely. Rotenstein et al. reported a burnout prevalence ranging from 0% to 80.5% among practitioners in 45 countries in Africa, Asia, Europe, the Middle East, North America, Oceania and South America.^11^ Chemali et al. found that 40%-60% of healthcare providers in the Middle East experienced burnout,^12^ and Dubale et al. identified high burnout levels among Sub-Saharan African healthcare professionals, especially nurses.^13^

The lives and health of patients are particularly important to professionals working in the medical field. Healthcare professionals’ burnout is highlighted due to their hectic schedule, demand for call duty and ability to work under pressure.^14^ In Malaysia, the staffing crisis exacerbates these pressures, leading to exhaustion and burnout among healthcare workers. Long working hours, limited time with family and inadequate compensation further contribute to burnout.

Addressing burnout requires a comprehensive understanding of both its contributing factors and the coping mechanisms employed by healthcare professionals. Effective coping strategies can mitigate the impact of stressors and enhance resilience among workers. Research suggests that problem-focused coping strategies such as seeking social support or engaging in physical activities, are associated with lower levels of burnout.^5,15^ Furthermore, fostering a supportive work environment and implementing targeted interventions can significantly enhance the well-being of healthcare workers.

This study is important not only for understanding the degree of burnout among healthcare professionals but also for exploring their preferred coping strategies to develop resilience within healthcare facilities. By identifying the factors contributing to burnout, this study can offer targeted interventions to mitigate stressors. Additionally, by exploring the coping mechanisms utilised by healthcare professionals, the study can highlight effective strategies for managing burnout and promoting resilience. Ultimately, this study has the potential to improve the well-being of healthcare professionals at Hospital Raja Permaisuri Bainun (HRPB), enhance the quality of patient care and contribute to the broader discourse on addressing burnout in the healthcare sector.

## Methods

### Study participants

This study employed an analytic observational method with a cross-sectional design, using online and printed questionnaires to collect data. Participants were recruited via non-probability convenience and snowball sampling. Invitations were disseminated via printed questionnaires and WhatsApp links. The study population comprised healthcare professionals from HRPB in Ipoh, Perak. HRPB has approximately 4202 staff members, of whom around 70% are healthcare professionals. A total of 382 healthcare professionals (approximately 13% of the workforce) completed the survey. Participants were selected if they were employed at HRPB, agreed to participate in the study, possessed proficiency in English, and were of Malaysian nationality. This study was approved by the Medical Research and Ethics Committee (NMRR ID-24-00041-NF5). Written informed consent was obtained from all study participants.

### Study setting

This study was conducted at HRPB, a tertiary care hospital that serves as a referral centre for various cases from across the state of Perak. As the most populous state in Malaysia, Perak relies on HRPB for its comprehensive range of specialised medical services and advanced healthcare facilities.

### Research instrument and scoring method

The questionnaire for this study was divided into four sections (A, B, C and D), totalling 39 questions:

1. Section A: Participant information and informed consent
2. Section B: Socio-demographic profile
  In this section, participants were asked about their socio-demographic details, including sex, age, marital status, professional position and educational level. Responses were collected through multiple-choice questions.
3. Section C: Prevalence of burnout
  Adapted from the Copenhagen Burnout Inventory (CBI),^5^ this section consisted of 17 questions aimed at assessing the prevalence of burnout among healthcare professionals at HRPB. The CBI measures burnout across three dimensions: personal burnout (five items), work-related burnout (six items) and patient-related burnout (six items). Respondents rate each item on a scale ranging from ‘always/to a very high degree’ (scored as 100%) to ‘never/to a very low degree’ (scored as 0%). According to Kristensen et al.^9^ and Borritz et al.,^16^ scores of 50-74 indicate moderate burnout; 75-99, high burnout; and 100, severe burnout.
4. Section D: Coping mechanisms against burnout
  This section, adapted from the Brief Coping Orientation to Problems Experienced Inventory (Brief-COPE),^17^ contained 12 questions to explore how healthcare professionals at HRPB cope with burnout. In the Brief-COPE, respondents indicate how often they employ various coping mechanisms on a 4-point Likert scale, ranging from 1 (‘I have not been doing this at all’) to 4 (‘I have been doing this a lot’). The coping strategies were categorised into two main styles:
  - Avoidant coping: This included selfdistraction, denial, substance use, behavioural disengagement, venting and self-blame.
  - Approach coping: This encompassed active coping, seeking emotional support, using informational support, positive reframing, planning and accepting.

Humour and religion were assessed due to their cultural relevance in Malaysia but not classified under either coping style. The total scores for each coping style were converted into percentages, where higher percentages indicated a greater reliance on that particular coping style.

### Content validation

Content validation was conducted to ensure the relevance, clarity and coherence of the questionnaire items. Two experts reviewed the questionnaire domains and items, rating their relevance on a 4-point scale. Items rated as 3 or 4 were considered relevant. The content validity index (CVI) for all items exceeded 0.80, meeting validity criteria, with a Scale-Content Validity Index/Average (S-CVI/Ave) of 0.99 and a Scale-level Content Validity Index (S-CVI/UA) of 0.98.

### Data analysis

Data were collected and tabulated in Microsoft Excel. They were then analysed using IBM SPSS Statistics for Windows, version 29 (IBM Corp., Armonk, N.Y., USA). Descriptive statistical analysis was used to examine the prevalence of mental and psychological burnout among healthcare professionals at HRPB and their socio-demographic profile. Pearson’s chi-square and inferential statistics were utilised to identify the association between the prevalence of burnout and socio-demographic factors (age, sex and educational level). A P-value of ≤0.05 was considered statistically significant.

## Results

### Socio-demographic profile

The study included 382 healthcare professionals from HRPB who completed the questionnaire distributed from late March to mid-April 2024. Among them, the significant majority were women (80.9%) and aged 25-35 years (55.8%). Most participants were married (69.4%), and the predominant professional group was nurses (41.4%), followed by doctors (22%) and pharmacists (14.4%). In terms of educational level, nearly half of the participants held a diploma (48.7%), with the remainder having bachelor’s (41.6%), master’s (9.4%) or PhD degrees (0.3%). Table 1 shows the participants’ socio-demographic profile.

**Table 1:** Socio-demographic profile of the participants (N=382).

Characteristic	Category	n (%)
Sex	Female	309 (80.9)
	Male	73 (19.1)
Age (year)	18-25	14 (3.7)
	25-35	213 (55.8)
	35-55	154 (40.3)
	>55	1 (0.3)
Marital status	Married	265 (69.4)
	Single	110 (28.8)
	Divorced	7 (1.8)
Professional position	Doctor	84 (22)
	Nurse	158 (41.4)
	Pharmacist	55 (14.4)
	Physiotherapist	15 (3.9)
	Medical assistant	20 (5.2)
	Pharmacy assistant	6 (1.6)
	Radiotherapist	3 (0.8)
	Others:	41 (10.8)
	Microbiologist	1 (0.3)
	Pathologist	2 (0.5)
	Medical laboratory technologist	32 (8.4)
	Dietitian	1 (1.6)
Educational level	PhD	1 (0.3)
	Master’s degree	36 (9.4)
	Bachelor’s degree	159 (41.6)
	Diploma	186 (48.7)

### Prevalence of burnout

The study examined burnout among 382 Malaysian healthcare professionals across three dimensions: personal burnout, work-related burnout and patient-related burnout using the CBI (Table 2). Among the participants, 54.7% experienced moderate burnout. Personal burnout was prevalent in 81.9%, work-related burnout in 81.9% and patient-related burnout in 85.5%.

**Table 2:** Prevalence of personal, work-related and patient-related burnout among the participants.

Total prevalence n (%)			
Socio-demographic characteristic	Personal burnout	Work-related burnout	Client (patient)-related burnout
Sex			
Female	256 (82.8)	254 (82.4)	272 (88.0)
Male	57 (78.1)	59 (80.8)	63 (86.3)
Age (year)			
18-25	9 (64.3)	12 (85.7)	12 (85.7)
25-35	169 (79.3)	169 (79.3)	186 (87.3)
35-55	134 (87.0)	131 (85.1)	136 (88.3)
>55	1 (100)	1 (100)	1 (100)
Marital status			
Married	218 (82.3)	219 (82.6)	230 (86.8)
Single	88 (80.0)	87 (79.1)	99 (90.0)
Divorced	7 (100)	7 (100)	6 (85.7)
Professional position			
Doctor	61 (72.6)	53 (63.1)	71 (84.5)
Nurse	135 (85.4)	139 (88.0)	139 (88.0)
Pharmacist	41 (74.5)	47 (85.5)	48 (87.3)
Physiotherapist	14 (93.3)	14 (93.3)	14 (93.3)
Medical assistant	18 (90.0)	18 (90.0)	17 (85.0)
Pharmacy assistant	5 (83.3)	4 (66.7)	5 (83.3)
Radiotherapist	2 (66.7)	1 (33.3)	2 (66.7)
Others:	37 (90.2)	37 (90.2)	39 (95.1)
Microbiologist	1 (100)	1 (100)	1 (100)
Pathologist	2 (100)	2 (100)	2 (100)
Medical laboratory technologist	28 (87.5)	28 (87.5)	31 (96.9)
Dietitian	6 (100)	6 (100)	5 (83.3)
Educational level			
PhD	1 (100)	1 (100)	1 (100)
Master’s degree	25 (69.4)	25 (69.4)	32 (88.9)
Bachelor’s degree	130 (81.8)	125 (78.6)	139 (87.4)
Diploma	157 (84.4)	162 (87.1)	163 (87.6)
**Overall**	313 (81.9)	313 (81.9)	335 (85.5)

### Coping mechanisms

Table 3 details the coping mechanisms implemented by the participants, assessed using 12 questions with four response options: ‘I have not been doing this at all’, ‘a little bit’, ‘a medium amount’ and ‘I have been doing this a lot’. For instance, 61 participants (16%) reported not using work or activities to distract themselves at all, while 135 (35.3%) did so a bit, 120 (31.4%) a medium amount and 66 (17.3%) often. Similarly, 343 participants (89.8%) did not use alcohol or drugs to feel better, while 14(3.7%) did so a bit, 17 (4.5%) a medium amount and 8 (2.1%) often.

**Table 3:** Coping mechanisms applied by the participants (N=382).

Coping mechanism	n (%)			
	I have not been doing this at all	A little bit	A medium amount	I have been doing this a lot
1. I have been turning to work or other activities to take my mind off things.	61 (16)	135 (35.3)	120 (31.4)	66 (17.3)
2. I have been using alcohol or other drugs to make myself feel better.	343 (89.8)	14 (3.7)	17 (4.5)	8 (2.1)
3. I have been getting emotional support from others.	82 (21.5)	135 (35.3)	118 (30.9)	47 (12.3)
4. I have been giving up trying to deal with it.	166 (43.5)	137 (35.9)	62 (16.2)	17 (4.5)
5. I have been taking action to try to make the situation better.	50 (13.1)	103 (27)	150 (39.3)	79 (20.7)
6. I have been refusing to believe that it has happened.	186 (48.7)	140 (36.6)	50 (13.1)	6 (1.6)
7. I have been saying things to let my unpleasant feelings escape.	130 (34)	147 (38.5)	81 (21.2)	24 (6.3)
8. I have been getting help and advice from other people.	74 (19.4)	153 (40.1)	104 (27.2)	51 (13.4)
9. I have been criticising myself.	157 (41.1)	137 (35.9)	67 (17.5)	21 (5.5)
10. I have been trying to come up with a strategy about what to do.	44 (11.5)	115 (30.1)	145 (38)	78 (20.4)
11. I have been looking for something good in what is happening.	44 (11.5)	80 (20.9)	148 (38.7)	110 (28.8)
12. I have been making jokes about it.	78 (20.4)	137 (35.9)	118 (30.9)	49 (12.8)

The coping strategies were categorised into avoidant, approach, humour and religious methods. Religion was the most utilised coping strategy, with 92.4% of the participants engaging in it, followed by approach coping (85.8%) and humour (79.6%). Avoidant coping was less common but included behaviours such as self-distraction (88.6%) and venting (69.8%). Strategies such as planning, acceptance and positive reframing were prominent within approach coping, with over 87% of the participants using these methods.

### Association between the socio-demographic profile and prevalence of burnout

Table 4 illustrates the association between the socio-demographic profile and the prevalence of burnout among the participants. This association was determined using the chi-square test. A P-value of less than 0.05 was considered statistically significant. The data were analysed and are presented in the table below.

**Table 4:** Association between the socio-demographic profile and prevalence of burnout among the participants (N=382).

	Severe n (%)	High n (%)	Moderate n (%)	Low n (%)	χ^2^ (df)	P-value
**Sex** Female Male	3 (1.0) 1 (1.4)	105 (34.0) 25 (34.2)	170 (55.0) 39 (53.4)	31 (10.0) 8 (11.0)	0.168 (3)	0.983
**Age (year)**					102.801 (9)	<0.001
18-25	0	2 (14.3)	11 (78.6)	1 (7.1)		
25-35	1 (0.5)	68 (31.9)	117 (54.9)	27 (12.7)		
35-55	2 (1.3)	60 (39.0)	81 (52.6)	11 (7.1)		
>55	1 (100)	0	0	0		
**Marital status**					8.607 (6)	0.197
Married	4 (1.5)	99 (37.4)	135 (50.9)	27 (10.2)		
Single	0	30 (73.4)	68 (61.8)	12 (10.9)		
Divorced	0	1 (14.3)	6 (85.7)	0		
**Professional position**					36.759 (30)	0.184
Doctor	1 (1.2)	20 (23.8)	45 (53.6)	18 (21.4)		
Nurse	3 (1.9)	53 (33.5)	90 (57.0)	12 (7.6)		
Pharmacist	0	17 (30.9)	34 (61.8)	4 (7.3)		
Physiotherapist	0	7 (46.7)	7 (46.7)	1 (6.7)		
Medical assistant	0	7 (35.0)	12 (60.0)	1 (5.0)		
Pharmacy assistant	0	3 (50.0)	3 (50.0)	0		
Radiotherapist	0	0	2 (66.7)	1 (33.3)		
Others:	0	21 (47.4)	18 (51.0)	2 (1.6)		
Microbiologist	0	1 (100)	0	0		
Pathologist	0	0	2 (100)	0		
Medical laboratory technologist	0	18 (56.3)	12 (37.5)	2 (6.3)		
Dietitian	0	2 (33.3)	4 (66.4)	0		
**Educational level**					8.256 (9)	0.509
PhD	0	0	1 (100)	0		
Master's degree	0	7 (19.4)	23 (63.9)	6 (16.7)		
Bachelor's degree	1 (0.1)	59 (37.1)	81 (50.9)	18 (113)		
Diploma	3 (1.6)	64 (34.4)	104 (55.9)	15 (8.1)		

There was a significant association between age (P<0.001) and the prevalence of burnout. However, sex (P=0.983), marital status (P=0.197), professional position (P=0.184) and educational level (P=0.509) exhibited no significant association with the prevalence of burnout.

## Discussion

### Prevalence of burnout

This study revealed that the healthcare professionals at HRPB experienced high levels of burnout, with 81.9% reporting personal and work-related burnout and 85.5% reporting client-related burnout. These findings align with those of the previous study conducted in Malaysia by Pang et al., showing a high prevalence of work-related burnout at 72%. However, this previous study noted slightly lower rates of personal (44%) and client-related (60%) burnout, both of which were categorised as moderate.^18^ This comparison highlights that while work-related burnout is consistently high across studies, variations in personal and client-related burnout levels may exist among different healthcare settings.

Our study found that burnout was particularly prevalent among the healthcare professionals at HRPB, with 88.0% of the 158 nurses and 85.5% of the 55 pharmacists experiencing high levels of burnout. In contrast, only 63.1% of the 84 doctors reported moderate levels of burnout. These results are consistent with the report by Carayon et al., wherein up to 54% of nurses and physicians experienced severe burnout.^19^ However, Maroco et al. reported different findings in Portugal, where 1262 nurses experienced moderate burnout levels, and 466 doctors had lower burnout levels, with 21.6% indicating tolerable burnout.^20^ Additionally, Ghahramani et al. noted that in Iran, 52% of pharmacists experienced moderate burnout, while 66% of 383 participating nurses and doctors faced burnout incidents.^21^ These variations highlight the differing prevalence and intensity of burnout across healthcare roles and geographical locations.

The organisational culture within HRPB may contribute significantly to the burnout level of healthcare professionals. A culture that lacks support for mental health, insufficient resources, and inadequate staffing can exacerbate stress among healthcare professionals. Studies have shown that positive organisational culture, characterised by supportive leadership and open communication, can mitigate burnout rates.^21^ Conversely, a toxic work environment can lead to increased emotional exhaustion and depersonalisation, as noted in a systematic review highlighting the role of workplace conditions in burnout prevalence.^8^

The high patient-to-staff ratios often experienced in healthcare settings can lead to overwhelming workloads for professionals, particularly nurses and pharmacists. This is consistent with findings from a study that indicated imbalances in duty allocation and resource constraints as significant contributors to burnout.^8^ The intense demands placed on healthcare workers during the COVID-19 pandemic have further intensified these issues, leading to higher emotional exhaustion rates.^22^

The disparity in the burnout levels between the nurses (88.0%) and pharmacists (85.5%) and the doctors (63.1%) can be analysed based on role-specific stressors and responsibilities. Nurses often bear the brunt of patient care responsibilities, leading to higher emotional labour demands. Research has indicated that nurses experience higher levels of emotional exhaustion due to their close interactions with patients and the emotional toll associated with caregiving.^8^

Pharmacists also face unique stressors related to medication management and patient counselling, which can contribute to client-related burnout. The increasing complexity of medication regimens and the pressure to ensure patient safety add layers of responsibility that may not be as pronounced in other roles. In contrast, doctors may experience different types of stressors related to decision-making and administrative burdens but often have more autonomy over their work schedules compared to nurses and pharmacists. This autonomy can serve as a buffer against burnout, although it does not eliminate the risk entirely.

The findings underscore the urgent need for targeted interventions aimed at reducing burnout among healthcare professionals, particularly nurses and pharmacists who are at a higher risk. Strategies could include improving organisational culture through supportive leadership training, implementing better staffing models to reduce workload pressures and providing mental health resources tailored for healthcare workers. Additionally, fostering a collaborative environment where all healthcare professionals can share their experiences may help mitigate feelings of isolation and stress.

In conclusion, while our study highlights alarming rates of burnout among healthcare professionals at HRPB, it also emphasises the importance of addressing systemic issues within the healthcare environment that contribute to this phenomenon. More effective strategies to support the well-being of the healthcare workforce can be developed by understanding the multifaceted nature of burnout and its varying impact across different roles.

### Coping mechanisms

The study revealed that the majority of healthcare professionals at HRPB used religion as a primary coping mechanism against psychological and mental burnout, with 92.4% relying on religious practices. Additionally, 85.8% employed approach coping styles, which included acceptance, active coping, positive reframing, planning and seeking of informational and emotional support. This aligns with the findings by Perez et al., who highlighted that relaxation techniques, including prayer and mindfulness, are effective in preventing burnout.^23^ Furthermore, our study found that 308 out of the 382 healthcare professionals (80.7%) used informational support as a significant coping strategy. This underscores the importance of approach coping, where informational support facilitates open discussions about mental challenges, thereby enhancing communication and empathy among team members and supervisors. Such practices help healthcare professionals manage emotions, acknowledge mistakes and express concerns, fostering a sense of community and reducing the isolation often felt in stressful situations.^24^

In our study, 87% of the participants utilised active coping, making it the second most likely approach used in coping with psychological burnout. A study conducted in Canada found that people who utilised active coping were less likely to experience burnout.^25^ Additionally, Mellins et al. found that individuals in surgical and anaesthesiology fields having professional training suited to their occupational environment and those participating in mental awareness campaigns were better equipped.^26^ In the present study, 78.5% of the participants reported emotional support as one of the most commonly used ways to mitigate the prevalence of burnout. This finding is supported by Boland et al., reporting that peer support stands out as a factor contributing to reducing burnout levels.^27^ In contrast, Howlett et al. suggested emotional approach styles as contributing factors to burnout.^25^ In our study, substance use as an avoidant coping style was less commonly utilised, with only 39% of the 382 participants using alcohol or drugs as a coping mechanism. Similarly, Guveli et al. reported that drug use was an ineffective coping method.^28^

### Association between the socio-demographic profile and prevalence of burnout

Our study reported that most healthcare professionals at HRPB (54.7%) experienced a moderate level of burnout. A significant association was found between sociodemographic profiles and the prevalence of burnout. Age, with a significance value of P<0.001, exhibited a significant association with the prevalence of burnout. Chou et al. similarly reported that young individuals were more likely to experience high levels of burnout.^29^ Additionally, Udho and Kabunga found that individuals below the age of 30 years exhibited a threefold higher risk of experiencing burnout in contrast to those aged 30 years and above.^30^ This pattern corresponds with the observed notion that burnout tends to diminish as age advances. A possible reason for this might be that younger individuals may also be more likely to take on additional responsibilities and work longer hours to show their commitment and dedication to their work in a hospital setting. This desire to excel and prove themselves could result in added work-related stress, ultimately leading to higher levels of burnout. Additionally, older professionals may experience lower burnout rates due to their years of experience, enabling them to identify effective coping strategies over time. This accumulated knowledge and ability to manage stressors contribute to their resilience and ability to balance work demands more effectively.

This study found that work-related burnout significantly correlated with professional position (P=0.013), highlighting that different healthcare professions experience varying levels of burnout. Chou et al. also reported that nurses and medical assistants were more prone to high burnout levels.^29^ Unexpectedly, our study revealed a substantial disparity in the burnout level between the doctors and medical assistants. Specifically, 41.6% (n=101) of the doctors reported burnout, compared to 70.2% (n=68) of the medical assistants. This aligns with the findings of Kuriyama et al., where approximately 30% of Japanese physicians experienced burnout, with nearly 40% considering leaving their current jobs or changing their career paths.^31^ Tur et al. also noted that medical professionals involved in surgical procedures were at an increased risk of mental health issues, leading to burnout.^32^ The differences in the burnout level among professions may be attributed to unique stressors: Doctors often face pressures from clinical decision-making and patient outcomes, nurses from direct patient care and medication administration and medical assistants from patient interactions, multitasking and administrative duties. Additionally, the organisational culture and support systems within healthcare settings likely differ across these professions, further influencing burnout prevalence.

The findings potentially confirm the hypothesis suggesting a connection between the socio-demographic profile, specifically age and professional position, and prevalence of burnout among healthcare professionals at HRPB. Despite the burnout level among these professionals appearing to be moderate, it is crucial for government organisations or institutions to persistently observe and comprehend the factors contributing to burnout.

## Conclusion

The study reveals moderate burnout among healthcare professionals at HRPB, with significant associations identified between their socio-demographic profile and burnout level. Notably, religion emerges as a prominent coping mechanism for managing burnout. However, the effectiveness of this coping strategy remains questionable, given the high prevalence of burnout in our study. This raises concerns about the appropriateness of the instruments used to assess coping mechanisms, suggesting that while religious coping is frequently employed, it may not sufficiently alleviate the stressors contributing to burnout. Furthermore, the study’s focus on a single hospital limits the generalisability of the findings to broader healthcare settings. While the insights gained are valuable for understanding burnout within HRPB, they may not reflect the experiences of healthcare professionals in other institutions or regions. Therefore, caution should be exercised when applying these results universally.

In light of the findings, it is essential for government institutions to consider implementing comprehensive training programmes and interventions tailored to address burnout effectively. Future research should explore the effectiveness of various coping strategies beyond those assessed in this study, as well as investigate burnout across different healthcare settings to provide a more nuanced understanding of this critical issue. Doing so can better support healthcare professionals in managing burnout and enhancing their overall well-being and job satisfaction.

### Study limitations

One limitation of this study is the use of single-item indicators to assess religion (‘I have been looking for something good in what is happening’) and humour (‘I have been making jokes about it’) as coping mechanisms. While these items provide insights into specific strategies, they fail to capture the multidimensional and complex nature of these constructs. Thus, the findings related to religion- and humour-based coping should be interpreted with caution. Future studies are recommended to incorporate multiple items or scales to ensure a more comprehensive and robust measurement of these coping mechanisms.

Additionally, the study was limited to healthcare professionals at HRPB due to time constraints, which may affect the generalisability of the findings. Nevertheless, the results are intended to provide insights applicable to broader healthcare settings in Malaysia. By identifying burnout factors and effective coping strategies, this research aims to contribute to the well-being of healthcare professionals at HRPB and inform approaches to mitigate burnout across the healthcare sector.

## Data sharing statement

The datasets generated and analysed during the current study are not publicly available due to ethical restrictions but are available from the corresponding author upon reasonable request.

**How does this paper make a difference in general practice?**

- This paper highlights the high prevalence of burnout among healthcare professionals in hospital settings, offering insights that are equally relevant to general practice.
- The findings underscore the critical need for targeted interventions, as burnout affects the wellbeing of healthcare providers and compromises the quality of patient care.
- Strategies such as integrating mental health support systems, promoting work-life balance and enhancing professional satisfaction are vital to addressing this issue.
- These insights can guide stakeholders, including policymakers and healthcare administrators, in developing effective measures to support healthcare professionals across various settings where similar pressures and demands are encountered.
